# Quality of life assessment in patients with heart failure: validity of the German version of the generic EQ-5D-5L™

**DOI:** 10.1186/s12889-019-7623-2

**Published:** 2019-11-06

**Authors:** Sigrid Boczor, Anne Daubmann, Marion Eisele, Eva Blozik, Martin Scherer

**Affiliations:** 1 Department of General Practice / Primary Care, Center for Psychosocial Medicine, University Medical Center Hamburg-Eppendorf, Germany, Martinistraße 52, 20246 Hamburg, Germany; 2 Department of Medical Biometry and Epidemiology, University Medical Center Hamburg-Eppendorf, Germany, Martinistraße 52, 20246 Hamburg, Germany

**Keywords:** Heart failure, Quality of life, EQ-5D-5L, Confirmatory factor analysis, Construct analysis, Discriminant validity

## Abstract

**Background:**

Chronic heart failure patients typically suffer from tremendous strain and are managed mainly in primary care. New care concepts adapted to the severity of heart failure are a challenge and need to consider health-related quality of life aspects. This is the first psychometric validation of the German EQ-5D-5L™ as a generic instrument for assessing health-related quality of life (HRQOL) in a primary care heart failure patient sample.

**Methods:**

Confirmatory factor analysis (CFA) was performed on the baseline EQ-5D-5L™ data from the RECODE-HF study (responses to all items from *n* = 3225 of 3778 patients). Basic CFA models for HRQOL were calculated based on the EQ-5D-5L™ items using the maximum likelihood (ML) and the asymptotic distribution-free method. In an extended CFA, physical activity and depression were added. The basic CFA ML model was verified for the reduced number of cases of the extended CFA model (*n* = 3064). In analyses of variance the association of the EQ-5D-5L™ visual analogue scale (VAS) and both the German and the British EQ-5D-5L™ crosswalk index with the SF-36 measure of general health were examined. The discriminant validity was analysed using Pearson’s chi-squared tests applying the New York Heart Association classification, for the VAS and indices analyses of variance were calculated.

**Results:**

In the basic CFA models the root mean square error of approximation was 0.095 with the ML method, and 0.081 with the asymptotic distribution-free method (Comparative Fit Index > 0.90 for both). Physical activity and depression were confirmed as influential factors in the extended model. The VAS and indices were strongly associated with the SF-36 measure of general health (partial eta-squared 0.525/0.454/0.481; all *p* <  0.001; *n* = 3155/3210/3210, respectively), also for physical activity and depression when included together (partial eta-squared 0.050, 0.200/0.047, 0.213/0.051 and 0.270; all *p* <  0.001; *n* = 3015/*n* = 3064/n = 3064, respectively). The discriminant validity analyses showed *p*-values < 0.001 and small to moderate effect sizes for all EQ-5D-5L™ items. Analyses of variance demonstrated moderate effect sizes for the VAS and indices (0.067/0.087/0.084; all *p* <  0.001; *n* = 3110/3171/3171).

**Conclusion:**

The German EQ-5D-5L™ is a suitable method for assessing HRQOL in heart failure patients.

## Background

Chronic heart failure (CHF) is a common disease managed mainly in primary care that typically places tremendous strain on patients in all developed countries [1–3]. The New York Heart Association (NYHA) classification is the standard approach to assessing severity; guidelines for the management of heart failure patients include the goal of improving health-related quality of life (HRQOL) [4–6]. Maximising HRQOL while treating CHF is still a major challenge [3, 6–9]. In primary care, CHF patients are in need of new concepts adapted to the severity of their disease that consider life expectancy and HRQOL. Patients with CHF mostly experience symptoms such as dyspnoea, fatigue, sleep disorders, and ankle oedema [10]. Several treatment strategies have been examined in the past decade with a view to improving HRQOL in CHF patients based on different influential factors. Using the Kansas City Cardiomyopathy Questionnaire (KCCQ), Whellan et al. 2007 identified physical exercise, the primary study objective in their randomised controlled HF-ACTION trial, as a ‘modest but statistically significant’ factor that influences HRQOL among CHF patients [11]. While this finding supported the pursuit of physical exercise in such patients [4–6], additional efforts were still necessary with respect to improving HRQOL, such as collecting sufficient data on end-of-life preferences related to HRQOL among elderly CHF patients [3, 8]. Stamp et al. 2014 studied CHF patients with NYHA class II and III in the context of family life, focusing specifically on patient autonomy – an aspect that so far has received little attention in studies [12].

Several treatment options are currently available to CHF patients which target either symptoms or prognosis, or both. It is questionable whether reduced mortality is the primary aim of treatment for CHF patients or whether patients would rather benefit from symptomatic relief, i.e. a better HRQOL [8]. Taking the baseline data of the TIME-CHF trial, Brunner-La Rocca et al. examined whether elderly CHF patients would prefer an improved HRQOL (e.g. no symptoms) over longevity [8]. They found that 74% of patients with a high burden of comorbidities were unwilling whereas 26% were willing to trade survival time for improved HRQOL [8]. The patients willing to trade longevity for quality of life were typically older, female, and living alone, and had a higher disease burden from CHF and a lower HRQOL as assessed by the Minnesota Living with Heart Failure questionnaire [8].

In 2012, Hoekstra et al. reported that most studies of CHF patients prefer to use a disease-specific rather than generic questionnaire to assess HRQOL [13]. However, a generic questionnaire permits HRQOL to be compared between patients with different diseases and therefore can add great value to research. Some larger, more specific questionnaires are available with which to evaluate the burden of heart failure [8, 14] but their completion demands a high level of willingness on the part of patients, which in the primary care setting is especially difficult. One of the world’s most widely used instruments for assessing HRQOL is the short generic EQ-5D®, which is available in more than 130 languages [15–17]. Clouth et al. tested the construct validity of the former 3-level German version of the EQ-5D® in CHF patients [16]. They found mixed evidence, with a low explained variance component and low factor loading on the question of anxiety and depression. The English version of the new 5-level EQ-5D® (EQ-5D-5L™) introduced in 2011 [18] has been validated in patients with cardiovascular disease, respiratory disease, depression, diabetes, liver disease, personality disorders, arthritis, and stroke [19]. However, worldwide validation of the EQ-5D-5L™, which expands the descriptive system response scale from three to five categories, is still ongoing. The EQ-5D-5L™ has not yet been validated for CHF patients. In general practice in particular, its time-saving application in the care of CHF patients should help to assess and thus improve HRQOL. In the research setting, moreover, use of the very short EQ-5D-5L™ could reduce the response burden. The goal of this research project was to validate the German version of the EQ-5D-5L™ for CHF patients.

### Key elements of the EQ-5D-5L™

The EQ-5D-5L™ consists of five dimensions (mobility, self-care, usual activities, pain/discomfort and anxiety/depression) related to a patient’s well-being (descriptive system; I1-I5). Each item can be answered on a 5-point scale (no/slight/moderate/severe/extreme problems). The answers to the five dimensions can be combined in a 5-digit number describing the patient’s state of health. Using a population-specific value set or EuroQol Groups’ crosswalk calculation [20], this number can be converted into an EQ index value which is commonly used to calculate quality-adjusted life years. The second patient self-rating measure is the EQ visual analogue scale (EQ VAS), whereby the well-being of the patient is rated with a number ranging from 0 (worst health the patient can imagine) to 100 (best health the patient can imagine). The EQ-5D® parameters analysed in this project comprised the aforementioned items, the EQ VAS, and the German and the British crosswalk index of the EQ-5D-5L™.

## Methods

To determine the suitability of the EQ-5D-5L™ for CHF patients, we evaluated its psychometric features using baseline data from the observational RECODE-HF study that has been described in detail in the study protocol and elsewhere [21–24]. In brief, ethical clearance has been granted by two local ethics committees. Patients with CHF had been recruited via German primary care practices between 2012 and 2014. Of the 4420 general practitioners (GP) invited, 293 finally participated. Patient inclusion criteria had been an informed consent, an age of at least 18 years, a CHF diagnosis documented within the last 5 years at the GP, and at the least one GP contact within the last 6 months; exclusion criteria were dementia and CHF patients who were not regular patients of the participating GP practice. Of 13,830 patients invited to participate in the RECODE-HF study, 5385 consented, and 4909 sent back the baseline questionnaire. The patient’s GP was interviewed by phone particularly regarding the patient’s comorbidities. The comorbidities of 3387 CHF patients were assessed. Of these patients the study population of the present analyses included all patients who answered the EQ-5D-5L™ descriptive system in full, i.e. *n* = 3225 patients, as well as thereof *n* = 3064/*n* = 3210/*n* = 3171 patients where the adjustment variables physical activity and psychosocial distress/the SF-36 general health status/the NYHA class were available, respectively. As the indices were calculated from the fully answered five items the sample size here was identical. Correspondingly, analyses of variance of the EQ-5D-5L™ VAS were based on patients with full data sets, i.e. the samples comprised of those patients who fully answered the five items and also answered the visual analogue scale as well as the included factors (physical activity and psychosocial distress: *n* = 3015; SF-36 general health status: *n* = 3155; NYHA class: *n* = 3110, respectively) as no data imputation algorithm was applied.

### Statistical analyses

Patient characteristics are presented as mean and standard deviation (SD) in the case of continuous data, and absolute and relative frequencies in the case of categorical data. Charlson’s Comorbidity Index was used to classify comorbid conditions [21]. CHF patients with co-morbid conditions were identified by Charlson’s comorbidities and compared to CHF only patients. On the EQ-5D-5L™ items Pearson’s chi-squared tests and for the EQ-5D-5L™ VAS and indices T-Tests were performed, respectively. The patients‘age was differentiated by quartiles and the EQ-5D-5L™ items were analysed by Pearson’s chi-squared tests, and for the EQ-5D-5L™ VAS and indices analyses of variance (ANOVA) was performed, respectively.

#### Construct validity

A confirmatory factor analysis (CFA) model was used to obtain evidence of one-dimensionality of the EQ-5D-5L™ items for mapping HRQOL in CHF patients [16]. Because of doubts about the multivariate normality, in addition to the maximum likelihood (ML) method the asymptotic distribution-free (ADF) method was applied to estimate the parameters [22, 23], and the results were compared. The estimates found to be quite similar (see Table 2), and this fact suggested that the multivariate normal distribution assumption was justified. Local fit measures (explained variance; average variance; correlation coefficient; standardised regression coefficient; factor reliability) [24, 25] and global fit measures (chi-squared statistic; root mean square error of approximation, RMSEA; Comparative Fit Index, CFI; Tucker-Lewis Index, TLI) [22, 25–27] were calculated.

To analyse the association with the question of general health on the SF-36, by rating general health on a five-point scale from ‘excellent’ to ‘poor’, analyses of variance (ANOVA) was performed for the EQ-5D-5L™ VAS and indices. The *P*-value and effect size (partial eta-squared) are presented; effect sizes were interpreted in accordance with Cohen [28, 29].

#### Extended CFA

The CFA model was extended by simultaneously adding a physical activity score and psychosocial distress assessment. In several studies, both parameters have been found to essentially influence HRQOL in CHF patients [5, 30, 31]. Psychosocial distress was classified according to a hierarchical algorithm [32], considering the Patient Health Questionnaire depression subscale (PHQ-9) [33–35], the Hospital Anxiety and Depression Scales (HADS-A and HADS-D) [36, 37], and the Patient-Reported Outcomes Measurement Information System (PROMIS®) depression and anxiety items [38, 39]. Parameter estimates are presented. To ensure reliable interpretation, the basic CFA ML model was recalculated with a reduced sample size due to the lower number of cases resulting from the model, including influential factors. The impact of physical activity and psychosocial distress on the EQ VAS measurement and indices was analysed using ANOVA. Physical activity and psychosocial distress were simultaneously added to each model. Model parameters are described.

CFA was performed with IBM SPSS Amos 23.0.0. Factor reliability and the average proportion of variance were calculated in accordance with M. Wirtz 2004 [24]. IBM SPSS Statistics 23.0.0.2 was used for all other calculations.

#### Internal consistency reliability

Cronbach’s coefficient alpha is given for the EQ-5D-5L™ items, as a typically reported measure for associations between multi-item scales. A limit of at least 0.90 for individual use was applied [40].

#### Discriminant validity

Pearson’s chi-squared tests were performed on the EQ-5D-5L™ items to examine the distribution of patients into the categories of heart failure as classified by the NYHA categories, the standard criterion for CHF severity [4–6]. The *p*-value is given, along with the Pearson correlation coefficient (interpreted as effect size after Cohen). ANOVA was performed to analyse the EQ-5D-5L™ VAS and indices and describe the association with the NYHA categories. The *P*-value and partial eta-squared are given.

## Results

Of all 3387 patients of the RECODE-HF study [32], 95% (*n* = 3225) completed the EQ-5D-5L™ descriptive system in full; of these, 98% (*n* = 3164) also completed the EQ-5D-5L™ VAS. Patient characteristics are listed in Table 1. The mean ± SD age of the patients was 74 ± 10 years, while the proportion of at least 80-year-olds was almost 30%. Of all patients 45% were female. Almost 50% of the patients had mild symptoms and a comparable number marked or no CHF symptoms or limitations, whereas only 3% had severe problems even at rest. Elderly patients ≥80 years accounted for almost 30%. Half of the patients were physically active on a daily basis. A total of 28% tended towards depression/depressive symptomatology/adjustment disorder or anxiety/anxiety disorder. Charlson’s Comorbidity Index could be calculated in 82% of patients and delivered a median index score of 2 (Q1: 1; Q3: 3) associated with a prediction of 10-year survival in these patients of 0.901.

**Table 1:** Baseline characteristics including comorbidities with prevalence greater than 20% in the patient sample

	All patients (*n* = 3225)
Female gender	1454 (45.1%)
Age (years)	73.9 ± 10.2
Education level (CASMIN)	
Primary	2040 (63.3%)
Secondary	847 (26.3%)
Tertiary	275 (8.5%)
Occupational status	
Employed	233 (7.2%)
Not employed	2931 (90.9%)
Living situation	
Living alone	970 (30.1%)
Together with others in private household	2123 (65.8%)
Living in an institution	62 (1.9%)
NYHA classification	
Class I	775 (24.0%)
Class II	1588 (49.2%)
Class III	721 (22.4%)
Class IV	87 (2.7%)
Missing	54 (1.7%)
Psychosocial distress	
No psychological disorder	1514 (46.9%)
Depression/depressive symptomatology/adjustment disorder likely	546 (16.9%)
Anxiety/anxiety disorder possible	370 (11.5%)
No psychological disorder likely	658 (20.4%)
None of these criteria applicable	137 (4.2%)
Cardiac decompensation/congestive heart failure with dyspnoea, improved during therapy	2374 (73.6%)
Arterial hypertension	1576 (48.9%)
Diabetes mellitus	1221 (37.9%)
Chronic ischaemic heart disease (also after myocardial infarction, ischaemic cardiomyopathy, angina pectoris)	1153 (35.8%)
Cardiac arrhythmias (atrioventricular block, cardiac arrest, paroxysmal tachycardia, atrial fibrillation)	952 (29.5%)
Kidney disease	913 (28.3%)
Dyslipidaemia	763 (23.7%)
Myocardial infarction	761 (23.6%)
Asthma/chronic pulmonary disease with pulmonary dyspnoea	708 (22.0%)

CASMIN, Comparative Analysis of Social Mobility in Industrial Nations (CASMIN criteria); NYHA, New York Heart Association; psychosocial distress classification according to hierarchical algorithm [32]; no more than 4% missing values per variable, except age and cardiac decompensation (both 5.5%); arterial hypertension, chronic ischaemic heart disease, cardiac arrhythmias and dyslipidaemia were optionally assessed with the International Statistical Classification of Diseases and Related Health Problems (ICD)-10 codes

Comparing CHF patients with co-morbid conditions to CHF only patients, statistically significant differences (*p* <  0.001) were presented by the EQ-5D-5L™ items mobility, usual activities and pain/discomfort as well as by the EQ-5D-5L™ VAS and indices. More of problems and lower VAS and index values were shown in the CHF patients with co-morbid conditions. Comparing age quartiles, all EQ-5D-5L™ items as well as the EQ-5D-5L™ VAS and indices presented statistically significant differences (p <  0.001) showing a deterioration of the patients’ conditions by higher age.

### Construct validity

**Fig. 1:**
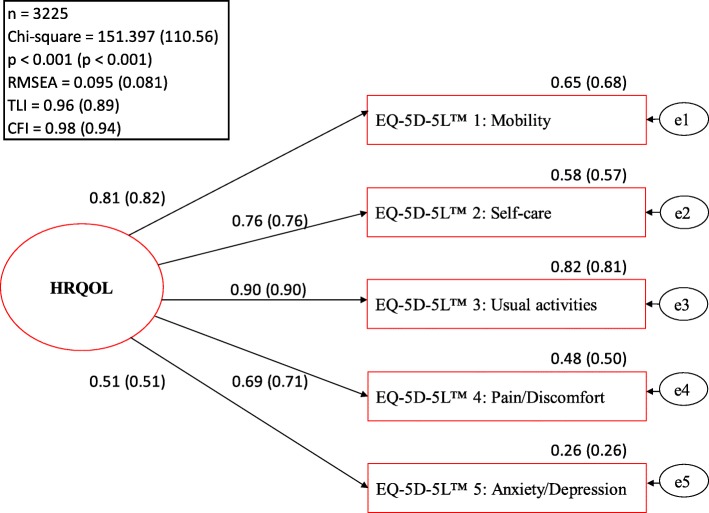
Health-related quality of life (HRQOL) represented by the EQ-5D-5 L™ items. Basic measurement model of the latent construct of HRQOL with model fit and standardised parameter estimates calculated using the maximum likelihood and asymptotic distribution-free methods (in parentheses). E1 - e5 = residual variation

**Fig. 2:**
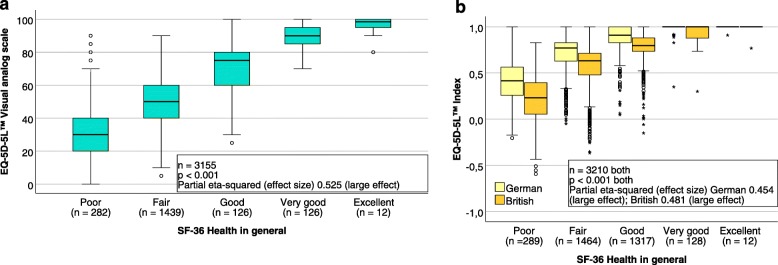
EQ-5D-5 L™ parameters and general health status. The association of the EQ-5D-5 L™ VAS (**a**), and the German and the British crosswalk index of the EQ-5D-5 L™ (**b**) with the SF-36 measure of general health

The basic CFA model is presented in Fig. 1*.* The standardised regression coefficients of the EQ-5D-5L™ items for the latent construct of HRQOL as well as the explained variances were almost identical when calculated with the ML and ADF methods. According to the local model fit statistics, the basic CFA model fit the data adequately. Only the explained variance of the item anxiety/depression revealed a value below the acceptable threshold. Reliability and the average proportion of variance were measured adequately by both methods; the global fit varied slightly (see Table 2). ANOVA results for the EQ-5D-5L™ VAS and the German and the British crosswalk index are presented in Fig. 2. Overall, strong associations were found between the EQ-5D-5L™ parameters and the respective SF-36 measure of general health (VAS: partial eta-squared 53%; German/British index 45%/48%).

**Table 2:** Measures of global fit, factor reliability and average proportion of variance for the basic CFA model and the CFA model adjusted for the variables physical activity and psychosocial distress

	Valid n	Chi^2^	Df	*P*-value	Chi^2^/Df	TLI	CFI	RMSEA	Factor reliability	AVE
Thresholds for model acceptance				> 0.05	< 3	≥ 0.90	≥ 0.90	≤ 0.08	> 0.60	> 0.50
*Basic model*										
Maximum likelihood method	3225	151.40	5	< 0.001	30.28	0.96	0.98	0.095	0.87	0.58
Asymptotic distribution-free	3225	110.56	5	< 0.001	22.11	0.89	0.94	0.081	0.87	0.59
*Adjusted model*										
Maximum likelihood method	3064	151.24	5	< 0.001	30.25	0.96	0.98	0.098	0.87	0.58
Asymptotic distribution-free	3064	109.87	5	< 0.001	21.98	0.88	0.94	0.083	0.87	0.59

CFA, confirmatory factor analysis; Df, degrees of freedom; TLI, Tucker Lewis Index; CFI, Comparative Fit Index; RMSEA, root mean square error of approximation; AVE, average proportion of variance measured; for thresholds see Schermelleh-Engel, Moosbrugger and Müller 2003; Kline 2011; Hooper, Coughlan and Mullen 2008 [22, 25, 26]

### Extended CFA

**Fig. 3:**
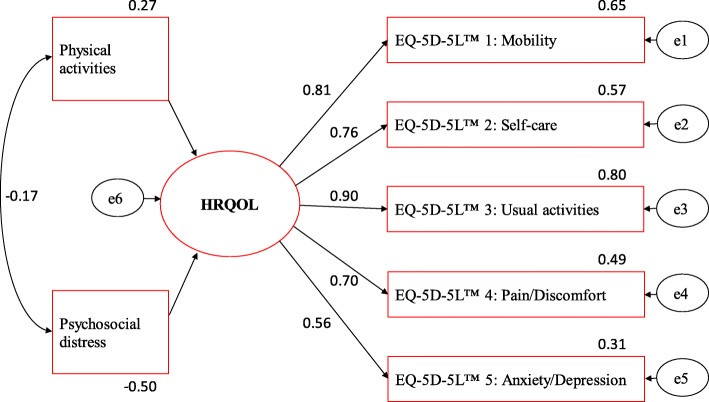
Essential influence factors on health-related quality of life (HRQOL) represented by the EQ-5D-5 L™ items. For the variables physical activity and psychosocial distress adjusted model of the latent construct of HRQOL with standardised parameter estimates calculated with the ML method. *N* = 3064; e1 - e6 = residual variation

**Fig. 4:**
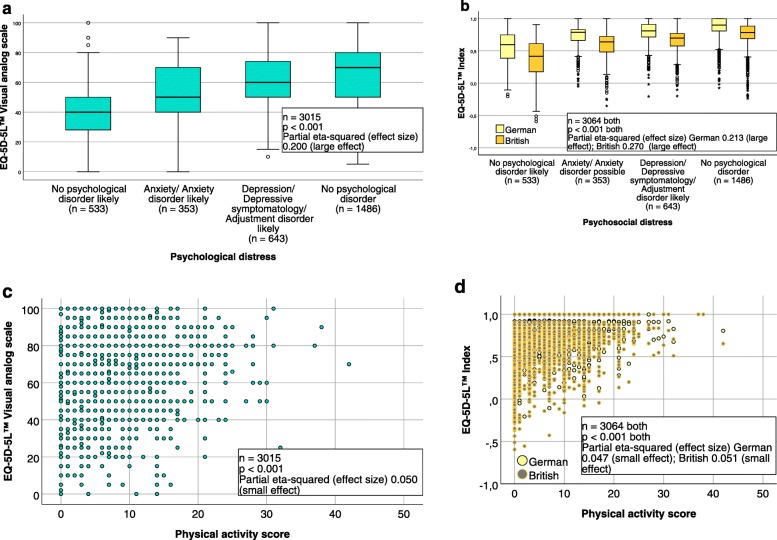
Essential influence factors on health-related quality of life represented by the EQ-5D-5L™ VAS and indices. The association of the EQ-5D-5L™ VAS (**a**) (**c**), and the German and the British crosswalk index of the EQ-5D-5L™ (**b**) (**d**) with physical activity and psychosocial distress

In the extended CFA, a small effect was found for physical activity, a strong negative effect for psychosocial distress, and a negative correlation between physical activity and psychosocial distress (see Fig. 3). ANOVA of the EQ-5D-5L™ VAS, the German and the British crosswalk index, including a physical activity score and psychosocial distress classification, proved statistically significant and confirmed the influence of these factors on HRQOL in our CHF patients (see Fig. 4).

### Internal consistency reliability

Cronbach’s coefficient alpha was 0.856, thus lying at the minimum threshold of 0.90 for individual use. A comparable value of 0.869 was obtained when experimentally removing the fourth item of anxiety/depression.

### Discriminative validity

**Fig. 5:**
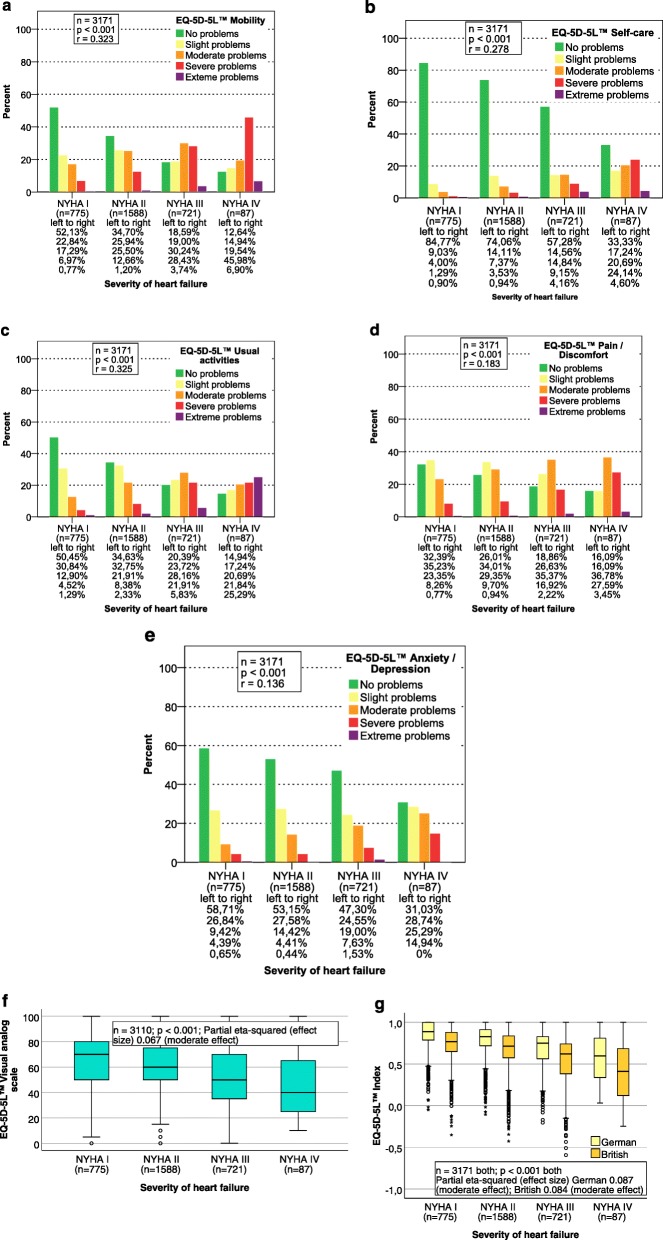
Discriminative ability indicated by the severity of heart failure according to the NYHA classes. The discriminative ability of the five EQ-5D-5L™ questions (**a**) (**b**) (**c**) (**d**) (**e**), the EQ-5D-5L™ VAS (**f**), and the German and the British EQ-5D-5L™ crosswalk index (**g**), respectively

The comparison between the NYHA categories and all five EQ-5D-5L™ items proved statistically significant (*p* <  0.001; effects I1/I3/VAS/indices = moderate; I2/I4/I5 = small; see Fig. 5). Few differences were found between NYHA I and II, the EQ-5D-5L™ VAS, and the German and the British crosswalk index. However, changes did become apparent in stages III and IV (see Fig. 5).

## Discussion

The EQ-5D-5L™ proved to be a reliable and valid method for measuring quality of life in CHF patients under primary care. The five questions of the short EQ-5D-5L™ were completed by *n* = 3225, i.e. 95%, of the primary care patients of the observational RECODE-HF study; of these, 98% also answered the EQ-5D-5L™ VAS. In the present study, the EQ-5D-5L™ was found to be a suitable short generic instrument for assessing CHF patients in the primary care setting. Reasonable discriminative and construct validity was found for all parameters of the EQ-5D-5L™. More of problems represented by the EQ-5D-5L™ items mobility, usual activities and pain/discomfort and lower VAS and index values were shown in the CHF patients with co-morbid conditions compared to CHF only patients, and also a higher age predicted deterioration shown by all EQ-5D-5L™ parameters. Thus giving evidence on the differences in the EQ-5D-5L™ parameters by CHF patients’ conditions. CHF patients are usually older patients and often have several comorbidities and risk factors, and typically the quality of life continually decreases as the severity of the disease increases [5]. Disease management guidelines also state that pathophysiological mechanisms of chronic heart failure and non-cardiac changes, e.g. musculoskeletal changes due to reduced blood flow, together explain the occurrence of exercise and stress intolerance, fatigue and lethargy in CHF patients [5, 6]. Only for the question on anxiety/depression was there an explained variance below the threshold in our analysis, as in the analyses by Clouth et al. with the former EQ-5D-3L™ in CHF patients [16]. Clouth et al. concluded that this resulted from the combination of two different definitions in the item anxiety/depression [16]. We agree and suggest, moreover, that due to the influence of psychosocial distress on other, physical areas, a depressive symptomatology is reflected in the response to all items. Physical activity and psychosocial distress were confirmed as influencing HRQOL in our research when measured with the EQ-5D-5L™ in CHF patients. In August 2017 the National Institute for Health and Care Excellence (NICE) in the UK released a statement on the EQ-5D-5L™, recommending use of the five-level version of the EQ-5D® for collecting data on HRQOL [41, 42]. We present values related to validity here that are necessary before widespread use of the tool in CHF patients.

Jaarsma et al. concluded in their qualitative study in CHF patients that psychosocial adaptation to disease could be enhanced by improving the ability to care for oneself [10]. As we have shown in Fig. 4, the disease burden is high in CHF patients with NYHA class III and IV. The degree to which self-care is restricted, as reflected by item 2 of the EQ-5D-5L™, was found to be an influential factor in CHF patients, and is assumed to be relevant to everyday life. Our findings support the conclusion of Stamp et al. 2014, namely that maintaining autonomy in patients with heart failure is of great importance [12].

Hoekstra et al. 2012 surmised that most CHF studies would rather use a disease-specific questionnaire to assess quality of life [13]. This could take the form of a specific application in clinical research, whereas in primary care there may be different priorities. Jaarsma et al. concluded that in clinical practice CHF patients cannot be easily compared to those in clinical trials or those undergoing transplant evaluation given that their functional capabilities are more greatly impaired [10]. However, according to McDonald et al. 2017 and the aspect of personalised care as the ideal future scenario, including high anticipated costs, use of a short generic questionnaire would seem more convenient given that time is limited in primary care consultations. When taking the medical history of a CHF patient, it is important to classify not only the heart failure burden but also, as this research highlights, activities of daily living and self-care, which is entirely feasible with the EQ-5D-5L™. This is crucial in CHF patients with a high disease burden. Brunner-La Rocca et al. demonstrated that patients with a higher disease burden were more willing to trade survival time for quality of life [8]. Our research with the generic EQ-5D-5L™ clearly distinguished CHF patients with severe heart failure of NYHA class III and IV from patients in a lower NYHA class with respect to HRQOL and CHF. Due to increased pain and physical discomfort as well as limited autonomy, HRQOL was severely limited in CHF patients with severe heart failure. This emphasises the special clinical relevance of these aspects.

To the best of our knowledge, our research is the first to validate the EQ-5D-5L™ for use in CHF patients. It can be assumed to offer additional benefit in the European context, not least because validation of the British version of the EQ-5D-5L™ for CHF patients is still pending. The extent of the overall burden of multimorbidity was comparable to the study of CHF patients in Italy by Gasperoni et al., which revealed a median Charlson Comorbidity Index score of 2 and identical quartiles (1; 3) to the Italian study patients who were hospitalised only once. Due to the shortness of the questionnaire its use in the primary care setting (coupled with patient decision management, and talking about the patient’s quality of life) may provide the impetus for discussion of the patient’s HRQOL. It could motivate patients to become more actively involved in improving their quality of life.

### Strengths and limitations

This is the first time that the EQ-5D-5L™ has been validated in a large sample of CHF patients in Germany.

One limitation of the RECODE-HF study is that patients were not primarily recruited with the aim of validating the EQ-5D®. As described in the study protocol, enrolment of patients with symptoms of anxiety/depression was prioritised [43]. However, the proportion of 28% CHF patients with possible/probable psychosocial distress is similar to the 30% depressive CHF patients in the German KCCQ validation study by Steinbüchel [44]. Similarly, in another German primary care study Scherer et al. reported a 29% increase in anxiety/depression in CHF patients [45]. Therefore, we assume that our CHF patient population is a representative sample and have described this sample thoroughly to allow for comparison of our results against other studies. For instance, the worsening of the CHF patients’ conditions by co-morbid conditions and age was shown by EQ-5D-5L™ items, the EQ VAS and indices, and also confirmed the representability of the sample [5].

Given that CHF is highly complex, our validation analyses did not include other factors that could affect HRQOL, such as age, co-medication, duration of disease, number of hospitalisations and patient adherence to therapeutic interventions. Whereas some measurement analyses with the EQ VAS of the EQ-5D-5L™ have been repeated using RECODE-HF follow-up data published recently [46], further research into the test-retest reliability of the EQ-5D-5L™ in CHF patients is still ongoing.

## Conclusion

The instrument is valid and reliable. This research into the EQ-5D-5L™ in CHF patients has shown that there is a strong correlation between HRQOL and health limitations that affect mobility and activities of daily living. They play a key role, especially in CHF patients with permanent health stress at advanced stages of the disease. In summary, it can be assumed that the very short generic German EQ-5D-5L™ is a suitable tool for assessing HRQOL in CHF patients in the primary care setting.

## Data Availability

The data from this study is not available for public use, as the data is owned by the RECODE-HF Study Group and the authors are not allowed to share this information with third parties.

## Abbreviations

ADF: Asymptotic distribution-free
ANOVA: Analyses of variance
CFA: Confirmatory factor analysis
CFI: Comparative Fit Index
CHF: Chronic heart failure
EQ-5D: Standardized instrument developed by the EuroQol Group as a measure of health-related quality of life
EQ-5D-5L: 5-level EQ-5D version
GP: General practitioner
HADS-A: Hospital Anxiety Scale
HADS-D: Hospital Depression Scale
HRQOL: Health-related quality of life
KCCQ: Kansas City Cardiomyopathy Questionnaire
ML: Maximum likelihood
NYHA: New York Heart Association
PHQ-9: Patient Health Questionnaire depression subscale
PROMIS®: Patient-Reported Outcomes Measurement Information System
RMSEA: root mean square error of approximation
SD: Standard deviation
TLI: Tucker-Lewis Index
VAS: Visual analogue scale
